# Carotid cavernous fistula with central retinal artery occlusion and Terson syndrome after mid-facial trauma

**DOI:** 10.3205/oc000063

**Published:** 2017-05-19

**Authors:** Satya Karna, Mukesh Jain, Md. Shahid Alam, Bipasha Mukherjee, Rajiv Raman

**Affiliations:** 1 Shri Bhagwan Mahavir Vitreoretinal Services, Sankara Nethralaya, Chennai, India; 2 Department of Orbit Oculoplasty Reconstructive and Aesthetic Services, Sankara Nethralaya, Chennai, India

**Keywords:** carotid cavernous fistula, central retinal artery occlusion, Terson syndrome, traumatic optic neuropathy

## Abstract

**Objectives:** To report a rare occurrence combination of central retinal artery occlusion (CRAO) and Terson syndrome in a Barrow’s type A carotid cavernous fistula (CCF) patient.

**Methods:** Observational case report.

**Results:** A twenty-year-old male patient with a history of road traffic accident presented with periorbital swelling and redness in the left eye. Examination revealed a CRAO with intraretinal and preretinal hemorrhages. On imaging, type A CCF and subarachnoid hemorrhage were detected. He underwent embolization of the fistula for cosmetic blemish. The possible mechanisms and clinical implications are discussed.

**Conclusion:** Patients with a head injury can have serious ocular damage. Posterior segment manifestations of CCFs are varied and at times can occur in various rare combinations, making it challenging. Early recognition of these rare manifestations and a multi-disciplinary approach are needed in patients with head trauma.

## Introduction

Carotid cavernous fistulas (CCFs) are an abnormal communication between the cavernous sinus and the carotid arterial system. The posterior segment manifestation of CCF are many, ranging from the most innocuous retinal vessels dilatation to sight-threatening traumatic optic neuropathy (TON), central retinal vein occlusion, glaucomatous optic neuropathy, and many others. Central retinal artery occlusion (CRAO) has rarely been reported in CCF patients in the past. There is only one case report in the literature by Pierre et al. of CRAO in traumatic CCF patient [1]. Pillai et al. reported a case of CRAO in dural CCF, in an old hypertensive (DSA) patient [2]. We report a case of type A CCF, confirmed on digital subtraction angiography, associated with CRAO and Terson syndrome. We discuss the possible mechanisms for our observation. 

## Case description

A twenty-year-old male presented with complaints of redness and swelling around the left eye of 2 weeks duration following a road traffic accident. Initial CT scan showed a small subarachnoid hemorrhage with a maxillofacial fracture. Open reduction and internal fixation (ORIF) was done for mandibular fracture. Plating and screwing were done for zygomatic fracture. MRI done subsequently showed dilated superior ophthalmic veins bilaterally and abnormally dilated vessels in the left cavernous sinus. Given the high suspicion of CCF, he was referred for ophthalmic evaluation.

**Figure 1:**
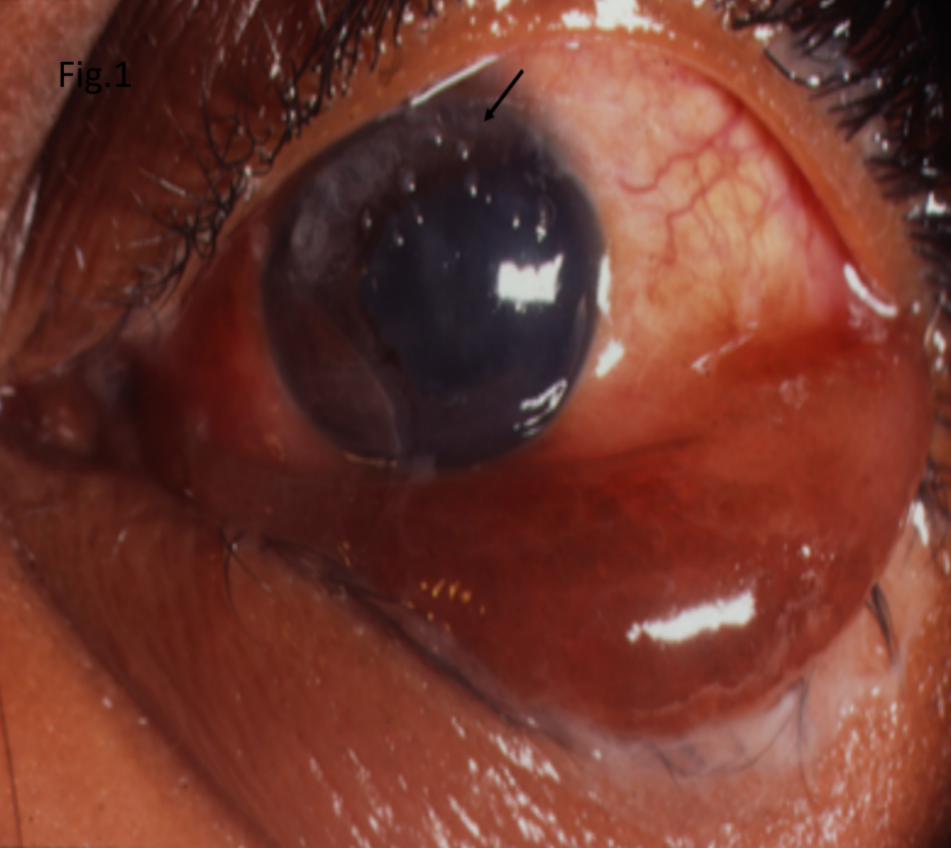
Slit lamp photograph of the left eye showing periorbital edema, conjunctival edema and chemosis, dilated cork-screw vessels and corneal mucous plaques (arrow) on the superior half of the cornea.

On examination, his best corrected visual acuity was 6/6 in the right and no light perception in the left eye. Extraocular movements were normal in the right eye but limited in all directions of gazes in the left eye. There were periorbital edema and near total ptosis in the left eye. Hertel’s exophthalmometry showed a reading of 20 mm and 27 mm in the right and left eye respectively with a base reading of 115. Slit lamp examination of the right eye showed a few prominent corkscrew subconjunctival vessels. In the left eye, there were conjunctival congestion and chemosis associated with dilated episcleral vessels, with few mucous plaques on the superior half of the cornea (Figure 1 (Fig. 1)). Pupillary reaction in the right eye was normal. The left eye had a 5 mm fixed and dilated pupil. Intraocular pressure was 22 mm Hg and 28 mm Hg in the right and left eye respectively by Goldman applanation tonometry. Fundus examination of the right eye revealed a normal disc with few intraretinal and sub-hyloid hemorrhages. In the left eye, the disc was mild hyperemic with attenuated arteries and dilated veins. There were a few intraretinal and preretinal hemorrhages with extensive whitening of the retina (suggestive of retinal edema) and a cherry red spot at the macula.

**Figure 2:**
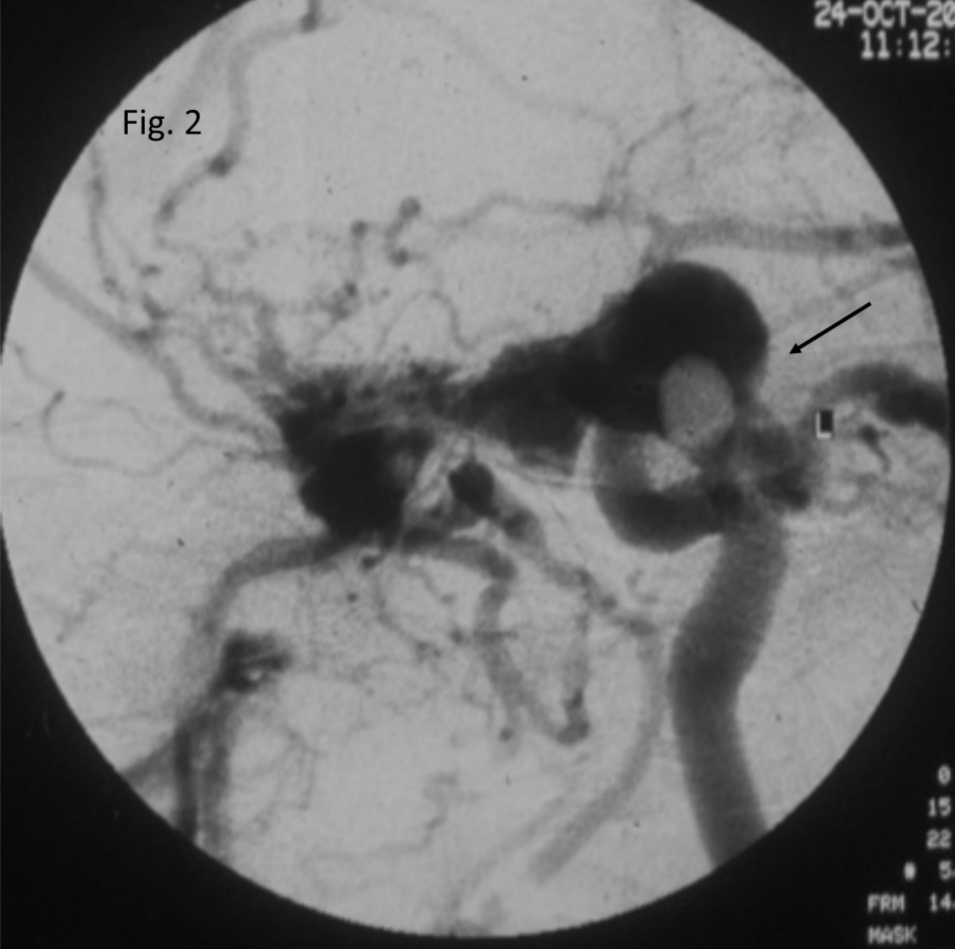
Digital subtraction angiography, antero-posterior view, left sided. Pooling of contrast material in the cavernous sinus with a direct communication (arrow) between the internal carotid artery and the cavernous sinus is seen, suggestive of Barrow’s type A carotid cavernous fistula.

A thrill could be felt on both sides while a bruit was noted only on the left side on palpation and auscultation respectively. Based on the clinical signs and symptoms, an initial diagnosis of CCF with recent CRAO was made. Digital subtraction angiography (DSA) was subsequently ordered. 4-vessel DSA revealed a high flow CCF Barrow’s type A (Figure 2 (Fig. 2)). Given the nil visual prognosis but considerable cosmetic blemish he underwent an attempt of embolization of the fistula but it was unsuccessful. 

**Figure 3:**
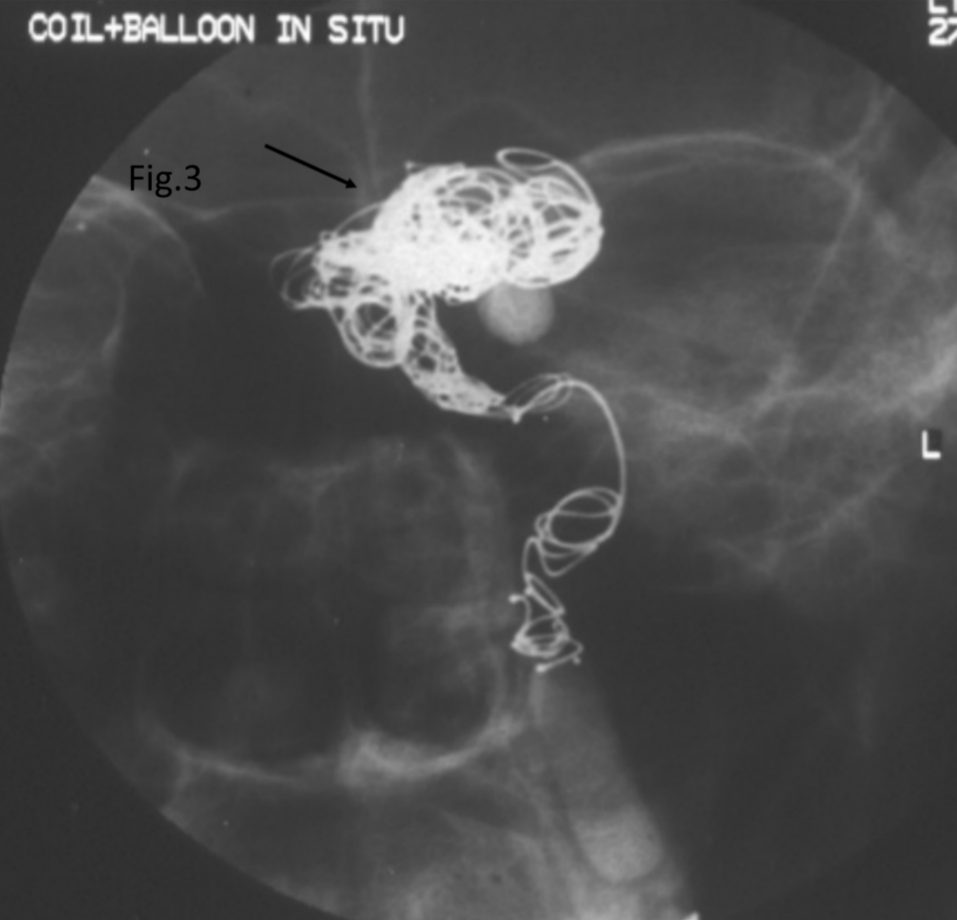
Digital subtraction angiography, lateral view, left sided. A coiling material and balloon (arrow) seen completely obliterating the fistula with no extravasation of the contrast agent. No feeding remnant is present.

At second visit one month later, the anterior segment examination of both eyes showed similar findings. Dilated fundus evaluation of the left eye showed optic atrophy with attenuated arterioles and mild pigmented pale looking retina. Two weeks later, he underwent a successful angioembolization (Figure 3 (Fig. 3)). Subsequently, the patient was lost to follow-up.

## Discussion

Patients with head injury can have serious ocular damage. A familiar periorbital swelling in the setting of trauma could be misleading, as in our case. A multi-disciplinary team approach is necessary for patients with head trauma. The posterior segment manifestations of CCF are varied, ranging from the most common innocuous finding of retinal vein dilatation to rare sight-threatening conditions as CRAO and Terson syndrome [1], [2], [3]. Since patients with head injury can sometimes present with various uncommon combinations of manifestations as in our case, early recognition and appropriate treatment can salvage vision.

TON is seen in 2–5% of facial traumas [4]. In our case, indirect type of TON can possibly be associated (CT scan showed no evidence of optic canal fracture). It results from shearing injury to the intracanalicular portion of the optic nerve, which causes traction and ischemic injury. There are rare case reports in literature of CRAO and traumatic optic neuropathy occurring following blunt trauma [5], [6]. 

Terson syndrome is the combination of intraocular (vitreous, sub-hyloid or intraretinal/sub-internal limiting membrane) hemorrhage associated with subarachnoid hemorrhage, intracerebral hemorrhage, or traumatic brain injury [7]. Terson syndrome occurs in 3.1% of traumatic head injuries [7]. Terson syndrome has been reported to be caused by or associated with multiple conditions related to a spike in intracranial pressure. A sudden rise in intracranial pressure results in rapid effusion of cerebrospinal fluid into the optic nerve sheath, resulting in dilation of the retrobulbar optic nerve. Consequently, the central retinal vein is mechanically compressed and causes venous hypertension causing rupture of thin retinal vessels. 

There could be multiple mechanisms for CRAO in CCF. First, an increased intraocular pressure secondary to raised orbital venous pressure could have led to CRAO [2]. The same mechanism is also responsible for Terson syndrome, which possibly explains their association in our patient. Second, a disruption of the vessels’ intimal layers by acute stretching in the setting of trauma damages the endothelium which could have led to thrombus formation [8]. Third, a local vasospasm in an injured vessel could have also contributed to the occlusion. Fourth, a lowered central retinal artery perfusion pressure from diversion of blood to venous system might be a cause of artery occlusion in our case.

In the previous case report by Pierre at al., the temporal association of CRAO and spontaneously closing traumatic fistula makes emboli the most probable etiology, unlike in our case [1]. There are multiple reports of CRAO complicating embolization attempts in CCF patients [9], [10], [11]. A fundus fluorescein angiography (FFA) could confirm the diagnosis of CRAO. But in the presence of altered ocular hemodynamics in CCFs patients, arm-retina time needs to be interpreted with caution. Moreover, on follow-up, attenuated arterioles with pigmented pale fundus supports our diagnosis.

## Conclusion

In conclusion, early recognition of this rare manifestation in CCF patients is important in the management of these patients. A multi-disciplinary team approach to a patient with head injury is essential for successful management. 

## Notes

### Acknowledgement

We would like to thank our colleagues in Shri Bhagwan Mahavir Vitreoretinal Services, Sankara Nethralaya, Chennai for their support.

### Competing interests

The authors declare that they have no competing interests.

## References

[1] Pierre Filho Pde T, Medina FM, Rodrigues FK, Carrera CR (2007). Central retinal artery occlusion associated with traumatic carotid cavernous fistula: case report. Arq Bras Oftalmol.

[2] Pillai GS, Ghose S, Singh N, Garodia VK, Puthassery R, Manjunatha NP (2002). Central retinal artery occlusion in dural carotid cavernous fistula. Retina (Philadelphia, Pa).

[3] Razeghinejad MR, Tehrani MJ (2011). Sudden onset and blinding spontaneous direct carotid-cavernous fistula. J Ophthalmic Vis Res.

[4] Holt GR, Holt JE (1983). Incidence of eye injuries in facial fractures: an analysis of 727 cases. Otolaryngol Head Neck Surg.

[5] Chong CC, Chang AA (2006). Traumatic optic nerve avulsion and central retinal artery occlusion following rugby injury. Clin Experiment Ophthalmol.

[6] Cumurcu T, Doganay S, Demirel S, Cankaya C (2011). Traumatic optic neuropathy and central retinal artery occlusion following blunt ocular trauma. J Clin Med Res.

[7] Czorlich P, Skevas C, Knospe V, Vettorazzi E, Richard G, Wagenfeld L, Westphal M, Regelsberger J (2015). Terson syndrome in subarachnoid hemorrhage, intracerebral hemorrhage, and traumatic brain injury. Neurosurg Rev.

[8] Scheerlinck TA, Van den Brande P (1994). Post-traumatic intima dissection and thrombosis of the external iliac artery in sportsman. Eur J Vasc Surg.

[9] Tan AC, Farooqui S, Li X, Tan YL, Cullen J, Lim W, Leng SL, Looi A, Tow S (2014). Ocular manifestations and the clinical course of carotid cavernous sinus fistulas in Asian patients. Orbit.

[10] Preechawat P, Narmkerd P, Jiarakongmun P, Poonyathalang A, Pongpech SM (2008). Dural carotid cavernous sinus fistula: ocular characteristics, endovascular management and clinical outcome. J Med Assoc Thai.

[11] Davis MC, Deveikis JP, Harrigan MR (2015). Clinical Presentation, Imaging, and Management of Complications due to Neurointerventional Procedures. Semin Intervent Radiol.

